# Transparent and Efficient Wood-Based Triboelectric Nanogenerators for Energy Harvesting and Self-Powered Sensing

**DOI:** 10.3390/polym16091208

**Published:** 2024-04-26

**Authors:** Ting Cheng, Kunli Cao, Yidan Jing, Hongyan Wang, Yan Wu

**Affiliations:** 1 College of Furnishings and Industrial Design, Nanjing Forestry University, Nanjing 210037, China; chengt@njfu.edu.cn (T.C.); jdjd2333@163.com (Y.J.); 2 Co-Innovation Center of Efficient Processing and Utilization of Forest Resources, Nanjing Forestry University, Nanjing 210037, China; 3 Academy of Forestry, Hangzhou 310023, China; 15990054143@163.com

**Keywords:** transparent TENG, wood based, epoxy resin, high performance

## Abstract

Wood possesses several advantageous qualities including innocuity, low cost, aesthetic appeal, and excellent biocompatibility, and its naturally abundant functional groups and diverse structural forms facilitate functionalization modification. As the most sustainable bio-based material, the combination of wood with triboelectric nanogenerators (TENGs) stands poised to significantly advance the cause of green sustainable production while mitigating the escalating challenges of energy consumption. However, the inherent weak polarizability of natural wood limits its development for TENGs. Herein, we present the pioneering development of a flexible transparent wood-based triboelectric nanogenerator (TW-TENG) combining excellent triboelectrical properties, optical properties, and wood aesthetics through sodium chlorite delignification and epoxy resin impregnation. Thanks to the strong electron-donating groups in the epoxy resin, the TW-TENG obtained an open-circuit voltage of up to ~127 V, marking a remarkable 530% enhancement compared to the original wood. Furthermore, durability and stability were substantiated through 10,000 working cycles. In addition, the introduction of epoxy resin and lignin removal endowed the TW-TENG with excellent optical characteristics, with optical transmittance of up to 88.8%, while preserving the unique texture and aesthetics of the wood completely. Finally, we show the application prospects of TW-TENGs in the fields of self-power supply, motion sensing, and smart home through the demonstration of a TW-TENG in the charging and discharging of capacitors and the output of electrical signals in different scenarios.

## 1. Introduction

With the rapid expansion of social production, there has been a concomitant increase in humanity’s energy requisites. However, the predominant energy sources are chiefly derived from fossil fuels such as coal, oil, and natural gas, exacerbating longstanding issues of resource depletion and environmental degradation [1,2,3]. Consequently, in recent years, the concepts of carbon emission reduction and sustainable development have been deeply rooted in people’s minds, and renewable energy and bio-based materials have attracted considerable attention. The functional modification of environmentally friendly materials, for example, to replace some of the current non-renewable and non-degradable polymerized materials, contributes to the realization of green and sustainable production and the alleviation of the growing problem of energy consumption [4,5,6].

Wood, as a natural polymer material, boasts a wide distribution, abundant stock, and a suite of advantages, including high strength, aesthetically pleasing texture, biodegradability, low price, and easy processing, and is widely applied in diverse sectors such as construction, home decoration, and agriculture. The unique three-dimensional layered structure, excellent porosity, and chemical composition of wood give it the opportunity to undergo functional modification [7,8,9]. Transparent wood (TW), obtained by removing the light-absorbing chromophores from natural wood and impregnating it with refractive index-matched polymers, offers a host of advantages including high transparency, low thermal conductivity, enhanced safety, light weight, environmental degradability, etc. [10,11,12]. Moreover, in order to meet the complex demands of social production, TW is being developed toward multifunctionality, with it poised to serve as a viable alternative to conventional materials such as metal, glass, and plastic [13,14,15].

Renewable energy is an effective solution and research focus in addressing the contemporary energy crisis, including wind energy, hydro energy, mechanical energy, and so on. Mechanical energy is a widely abundant, low-frequency, and irregular green energy in daily life, but it has not been effectively utilized [16,17,18]. Emerging as a sustainable energy-harvesting technology, triboelectric nanogenerators (TENGs), which are based on contract initiation and electrostatic induction between two materials with opposite polarity, can effectively convert mechanical energy into electric energy [19,20,21]. Compared with other energy-harvesting technologies, distinguished by their versatility, cost-effectiveness, and facile fabrication, TENGs find application across a spectrum of domains including self-powered sensors, medical instrumentation, and smart home appliances [22,23,24]. However, traditional TENGs are often composed of metals that are easily corroded or non-biodegradable synthetic polymers, which limits the scope of application of TENGs. Consequently, non-toxic and sustainable wood and its functionalized derivatives have become promising alternative materials [25,26,27].

In recent years, several wood-based TENGs have emerged in academia. Due to the weak polarity of natural wood, Luo et al. (2019) transformed natural wood into a high-performance tribomaterial with excellent strength through delignification and hot-pressing treatments, and the assembled W-TENGs showed a 70% increase in electrical output performance compared to logs, culminating in the development of a self-powered sensing table for motion data analysis [28]. Liao et al. (2023) improved the triboelectric properties of wood by introducing functional groups (amino groups) with improved electron transfer ability into wood, obtained an open-circuit voltage (Voc) of ~32 V and a short-circuit current (Isc) of 0.34 μA, and combined wood-based TENGs with home smart applications for sensing and energy supply roles [29]. However, most of these wood-based TENGs focused on enhancing electrical output performance while neglecting other aspects of functional innovation.

In this study, we report on a simple top-down approach to fabricating transparent wood-based triboelectric nanogenerators (TW-TENGs), which have high triboelectrical properties and excellent light transmittance, retaining the natural aesthetic texture of wood. By treating the natural wood with different degrees of delignification to increase the wood’s porosity while removing the chromophore groups, we lay the foundation for the introduction of epoxy resins enriched with strong electron-donating groups (amino and methyl groups). In addition, we characterized the micromorphology, chemical composition, optical properties, and mechanical properties of natural wood (NW), delignified wood (DW), and transparent wood (TW), thereby elucidating the impact of delignification extent on TW-TENG performance. The electrical output performance of TW-TENGs was tested to study the effect of different working conditions on it and to assess the stability of TW-TENGs in practical applications. This study presents for the first time an effective process for the fabrication of high-performance and highly transparent aesthetic wood-based triboelectric nanogenerators, which will have a profound impact on renewable energy, smart sensing systems, and smart decorative materials.

## 2. Materials and Methods

### 2.1. Materials

Maple (*Acer* spp.), size 40 mm × 40 mm × 0.5 mm (L × W × H), purchased from Deqing Meilun Decoration Material Co. Ltd. (Huzhou, China) Sodium chlorite (NaCIO_2_, 80%) and glacial acetic acid (CH_3_COOH, 99.5%) were purchased from Shanghai McLean Biochemical Technology Co. (Shanghai, China) Anhydrous ethanol (C_2_H_5_OH, ≥99.7%) was purchased from Sinopharm Group Chemical Reagent Co. (Beijing, China) Epoxy resin (H758) with an amino group content of 7.16 mmol/g was purchased from Dongguan Jinyuan New Material Co. (Dongguan, China) PDMS films were purchased from Hangzhou Wisdom Technology Co. (Hangzhou, China) ITO/FEP films were purchased from Shenzhen South China Xiangcheng Technology Co. Ltd. (Shenzhen, China).

### 2.2. Fabrication of TW-TENG

**Figure 1:**
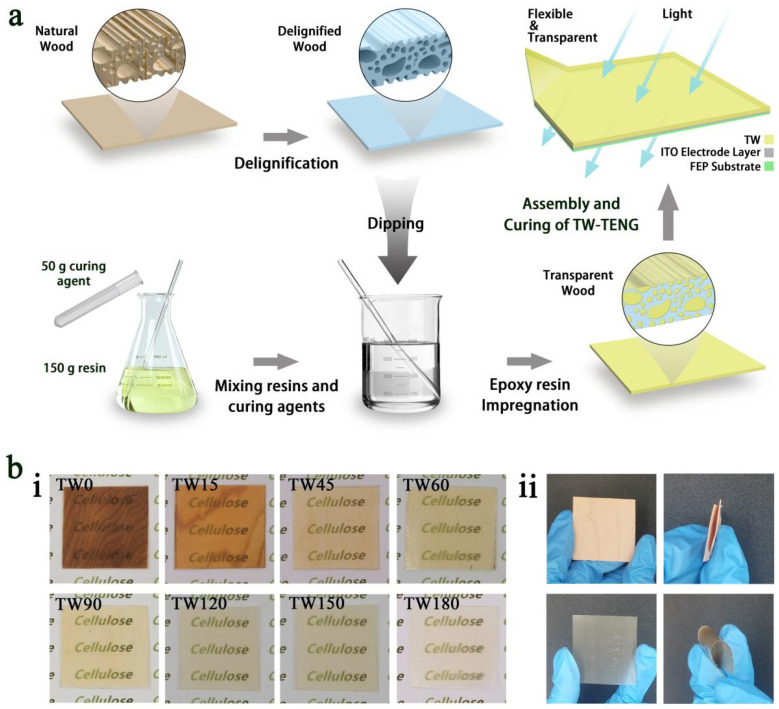
(**a**) Fabrication process of the TW-TENG; (**b**) **i**:Optical photographs of TW with different delignification time, **ii**: Flexibility of DW vs TW.

Initially, the natural wood (NW) was dried in a constant-temperature blast drying oven at 103 °C for 24 h. Then, delignification treatment was carried out by immersing the oven-dried maple wood in a mixed solution of ultrapure water and sodium chlorite (3.8 wt%) and adjusting the pH of the solution to 4.6 by adding acetic acid, followed by heating the solution in a water bath at 85 °C for different periods of time (0, 15, 45, 60, 90, 120, 150, and 180 min). Subsequently, the samples were rinsed with ultrapure water for 15 min to remove the chemical residues on the sample surface to obtain delignified wood DW) with different degrees of delignification (*n* = 15, 45, 60, 90, 120, 150, and 180). The sodium chlorite method was effective in removing the lignin and extractives of the wood, while cellulose and lignin were largely retained. Following delignification, the delignified substrates were soaked in ethanol for 24 h to displace the residual water in DW for the preparation of transparent wood. Subsequent to weighing and stirring the epoxy resin and curing agent in accordance with a mass ratio of 3:1, the DW was immersed in the epoxy resin and treated via vacuum impregnation for 3 h. Afterward, the vacuum pump was turned off, and the samples were taken out and assembled with the electrode layer of ITO/FEP films. Finally, a dual-layer glass plate served as a mold to clamp the assembled TENG device and wait for its finalization and solidification to obtain the TW*n*-TENG devices with different degrees of delignification (*n* = 0, 15, 45, 60, 90, 120, 150, and 180). Figure 1 shows the complete fabrication process and the finished samples.

### 2.3. Scanning Electron Microscopy

The dried samples were cut along the transverse direction using an ultramicrotome, and the samples were attached to the observation platform using conductive tape and sprayed with gold particles. The cross-sections of the samples perpendicular to the direction of wood fiber alignment before and after delignification and impregnation treatments were then observed using a Quanta 200 environmental scanning electron microscope (FEI, Hillsboro, OR, USA) at a pressurized electrical speed of 20 kV.

### 2.4. Fourier-Transform Infrared Analysis

The samples were completely dried before testing, and the attenuated total reflection (ATR) Fourier-transform infrared (FT-IR) spectra of NW, DW, and TW were measured using a VERTEX 80V spectrometer (BRUKER, Lücken, German). The samples were placed into the test chamber of the instrument and pressed with an ATR indenter to observe the changes in the vibrational peaks of the chemical groups in light transmission at wavenumbers from 500 to 4000 cm^−1^ for the samples before and after delignification and impregnation treatments. The sample size was 20 mm × 20 mm × 0.5 mm (L × W × H).

### 2.5. Chemical Composition Content Analysis

The relative content of cellulose, hemicellulose, and lignin in the NW and DW samples was examined according to the laboratory analytical procedure (LAP) (Determination of Structural Carbohydrates and Lignin in Biomass) prepared by the National Renewable Energy Laboratory (NREL).

### 2.6. Optical Properties

The optical transmittance of the samples was tested under visible light at a wavelength of 350~800 nm using a U3900 UV-visible spectrophotometer, and the haze value of the samples was calculated.

### 2.7. Mechanical Properties

The tensile strengths of the NW, DW, and TW samples were tested using a Shimadzu tensile testing machine (AGS-X 10KN, Kyoto City, Japan) with a gauge length of 40 mm, a tensile speed of 3 mm/min, and sample dimensions of 70 mm × 10 mm × 0.5 mm (L × W × H), and seven parallel samples were set up for each group of specimens and averaged with the results of the tests.

### 2.8. Electrical Output Performance

The single-electrode TW-TENG was fixed on the pedestal of the tensile tester, the PDMS film adhered to the upper press of the tensile tester, and then, the tensile tester was operated to drive the TW-TENGs at a fixed frequency and pressure. By connecting an electrostatic meter (Keithley 6514, Johnston, IA, USA) to one end of the TW-TENG and a computer to the other, the electrical signal generated by the TW-TENGs could be captured and output by the computer.

## 3. Results and Discussion

### 3.1. Scanning Electron Microscopy

**Figure 2:**
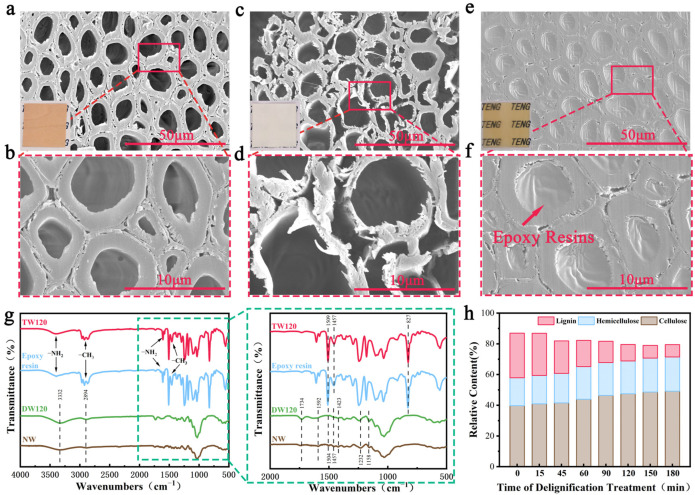
Morphological and chemical compositional characterization of NW before and after treatment. (**a**,**b**) Cross-sectional SEM images of NW; (**c**,**d**) cross-sectional SEM images of DW120; (**e**,**f**) cross-sectional SEM images of TW120. (**g**) FTIR infrared spectra of NW, DW120, and TW120 and magnified images within the dashed line. (**h**) Relative contents of cellulose, hemicellulose, and lignin in DW with different degrees of delignification treated using the sodium chlorite method.

Delignification treatment and resin impregnation play a crucial role in enhancing the electrical output performance and optical transmittance of natural maple wood. SEM images of natural maple (NW), delignified wood (DW), and transparent wood (TW) are shown in Figure 2. The parenchyma cells of NW were neatly arranged with rounded and regular shapes, presenting the unique three-dimensional porous structure of wood (Figure 2a,b). The cell walls of the wood treated with 120 min delignification (DW120) produced slight deformation, and the removal of the lignin and extractives resulted in wider gaps and delamination between the cells, but the cell channels were largely preserved, providing sufficient channels and space for resin introduction (Figure 2c,d) [30,31,32]. However, when the delignification duration exceeded 120 min, the SEM images of TW150 and TW180 showed significant fragmentation of the cell walls and obvious cracks between the cell walls and between the cell walls and the epoxy resin, suggesting that delignification for an excessively long period of time disrupts the integrity of the cellulose skeleton, which, in turn, affects the compatibility of the resin with DW (Figure S1a–c). After the DW was successfully impregnated with epoxy resin enriched with electron-donating groups (amino and methyl groups), its cell cavities were filled with epoxy resin, and the weakly polar air that originally existed in the internal pores was expelled, which helped to improve the triboelectric properties of the wood (Figure 2e,f). Meanwhile, the color change of the samples in the internal insets of Figure 2a,c,e and the improvement in transparency reflected the lignin removal and impregnation of the epoxy resin.

### 3.2. Fourier-Transform Infrared Analysis

Fourier-transform infrared spectroscopy (FTIR) analysis of NW, DW, and TW was performed as shown in Figure 2g. In agreement with previous studies [33,34,35], NW exhibited characteristic peaks at wavenumbers of 3332 cm^−1^ (O-H stretching vibration), 1734 cm^−1^ (C=O stretching vibration), 1592 cm^−1^ (carbonyl stretching vibration in lignin), 1504 cm^−1^ (aromatic skeletal vibration in lignin), 1457 cm^−1^ (C-H bending vibration in lignin and polysaccharides), 1423 cm^−1^ (cellulose CH_2_ shear vibration), 1232 cm^−1^ (C-O stretching vibration in acetyl O=C-O), 1158 cm^−1^ (C-O-C stretching vibration), and others. The characteristic peaks of DW120 treated with sodium chlorite delignification disappeared or narrowed at wavenumbers of 1592 cm^−1^, 1504 cm^−1^, 1457 cm^−1^, 1423 cm^−1^, and 1232 cm^−1^, and the acetyl group stretching vibration peaks in hemicellulose and C-O-C stretching vibration peaks in cellulose appeared at 1734 cm^−1^ and 1158 cm^−1^, respectively, which proved that lignin was successfully removed and a large amount of cellulose and hemicellulose was retained [36]. Meanwhile, the Fourier-transform infrared spectroscopy (FTIR) diagrams of the TW presented the characteristic peaks of epoxy resin, i.e., 1509 cm^−1^ (bending vibration of the -C=C- para-substituted benzene ring), 1457 cm^−1^ (asymmetric vibrational band of the benzene ring skeleton), 827 cm^−1^ (face deformation peaks of the para-substituted benzene ring C=O), 1606 cm^−1^, 3399 cm^−1^ (amino stretching vibration), 1461 cm^−1^, and 2825 cm^−1^ (methyl stretching vibration), proving that epoxy resin was successfully impregnated into DW under vacuum conditions [37].

### 3.3. Chemical Composition Content Analysis

To further study the relationship between delignification duration and the degree of delignification, we characterized the relative contents of cellulose, hemicellulose, and lignin in the different samples. The wood is mainly composed of cellulose, hemicellulose, and lignin, and the compositional changes in the DW treated using the sodium chlorite method are shown in Figure 2h. The sodium chlorite solution had the greatest effect on lignin, and the relative lignin contents of NW, DW45, DW90, and DW150 were 29.1%, 21.2%, 13.9%, and 8.3%, respectively. The data reveal a proportional decrease in the relative content of lignin within the wood as the duration of delignification increases, concomitant with a corresponding augmentation in the relative content of cellulose and hemicellulose, proving that the sodium chlorite method successfully removed the lignin which hinders triboelectricity in wood.

### 3.4. Optical Properties

**Figure 3:**
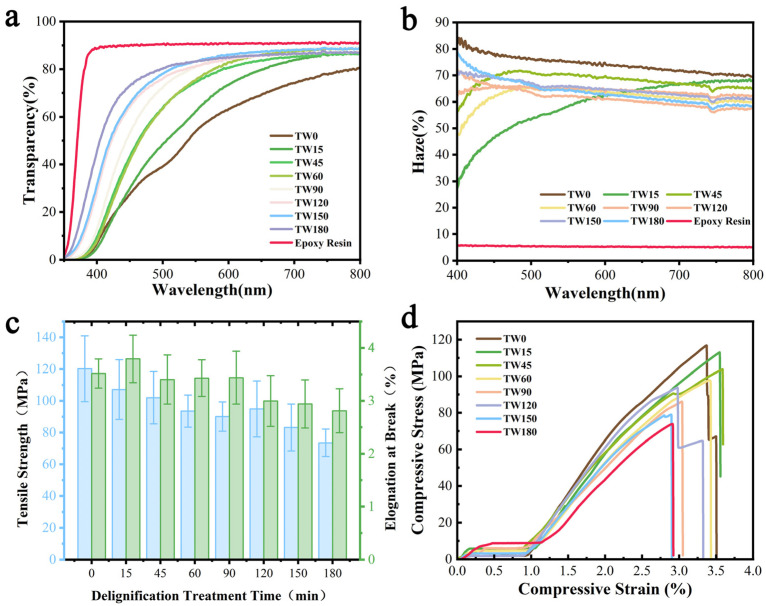
Characterization of the optical and mechanical properties of TW with different degrees of delignification. (**a**) Optical transmittance of TW with different degrees of delignification. (**b**) Haze of TW with different degrees of delignification. (**c**) Tensile strength and elongation at break of TW with different degrees of delignification. (**d**) Stress–strain curves of TW with different degrees of delignification.

Sodium chlorite delignification can effectively delignify lignin in wood to reduce light attenuation due to UV absorption by lignin, and at the same time, we can construct nanoporous wood to lay the foundation for the preparation of TW; as a result, the different delignification durations directly affect the light transmittance and haze of TW. The characterization of light transmittance and haze of different TW is shown in Figure 3a. The transparent wood samples impregnated with epoxy resin all showed high transparency. The transmittance of TW made from NW (TW0) reached 80.2%, the transmittance of TW treated with 15 min delignification (TW15) grew to 86.7%, and the transmittance values of TW45, TW60, TW90, TW120, TW150, and TW180 were 86.7%, 88.9%, 89.3%, 87.3%, 88.7%, and 87.1%, respectively. The results showed that the degree of delignification was positively correlated with the transmittance of TW in the range of 0~90 min, and the transmittance slightly decreased and fluctuated in the range of 120~180 min, which may be attributed to the fact that too much delignification time destroys the cellulosic skeleton of the wood, which negatively affects the compatibility of epoxy resin with wood templates [38]. Figure 3b demonstrates the haze of different TW, with a value of 69.0% for TW0, gradually decreasing to 67.8%, 65.77%, and 60.56% for TW15, TW45, and TW60, and decreasing to a minimum of 57.3% for TW90, followed by a slight increase to 62.4%, 61.4% and 58.6% for TW120, TW150 and TW180, respectively. Between the delignification time of 0 and 90 min, the haze of TW exhibited a decremental trend, correlating with the treatment duration, while the haze of TW increased slightly beyond a delignification duration exceeding 90 min. In summary, among the TW with different delignification levels, TW90 had the highest light transmission of 89.3% and the lowest haze of 57.3%.

Combined with the macrophotographs (Figure 1b(i)), the TW impregnated with epoxy resin all possessed extremely high transparency, the visualization of the samples was consistent with the light transmission test data, and the TW all preserved the aesthetic texture of natural wood completely and showed an amber texture due to the epoxy resin, with them featuring highly practical aesthetic decorative properties. Meanwhile, from Figure 1b(ii), it can be seen that the introduction of epoxy resin makes the TW more flexible, which provides the possibility of practical application.

### 3.5. Mechanical Properties

Material strength is one of the key factors that determines the utility and durability of TW-TENGs, so we investigated the tensile strength, elongation at break, and stress–strain curves of TW with different degrees of delignification to more accurately assess the TW-TENGs (Figure 3c). Lignin content and impregnating resin content are the key factors that affect mechanical properties. After delignification treatment, the tensile strength of DW continued to show a decreasing trend, the cell walls of DW were severely damaged and the mechanical properties significantly declined after 90 min (Figure S2), and the tensile strength of DW180 was only ~10 MPa, which can be attributed to the fact that too long a delignification time destroys the integrity of wood templates [39]. The mechanical properties of the TW increased significantly, with the tensile strength of TW180 increasing to ~73 MPa and that of the other samples reaching up to ~120 MPa. Meanwhile, the elongation at the break of TW became significantly larger compared to DW, and the flexibility was improved, as evidenced by the stress–strain curves of the TW (Figure 3d and Figure S3). Overall, the mechanical properties of epoxy resin-impregnated TW were generally optimized with stronger tensile strength and better flexibility, which ensures its practical applications in the fields of wearable sensors and smart decorative materials.

### 3.6. Electrical Output Performance

**Figure 4:**
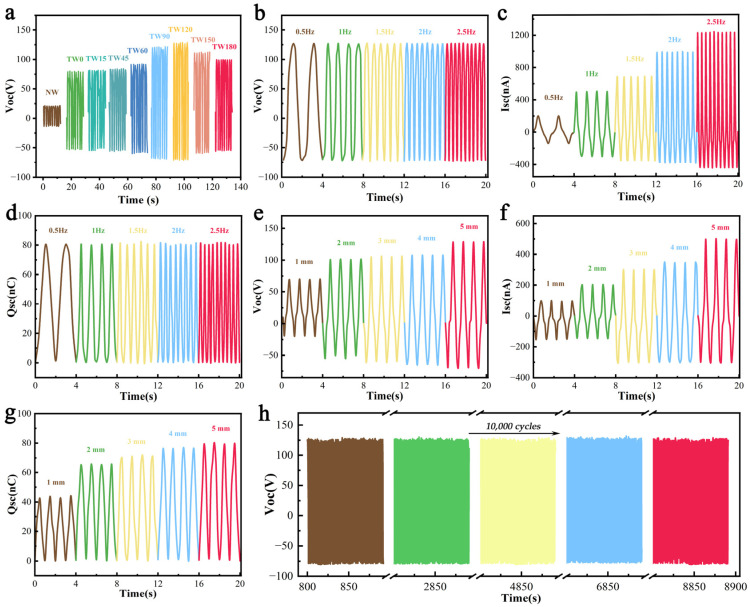
Electrical output performance tests for the TW-TENGs. (**a**) Triboelectrical performance Voc versus delignification treatment length. (**b**–**d**) Open-circuit voltage (Voc), short-circuit current (Isc), and transfer charge (Qsc) of TW120-TENG at different working frequencies. (**e**–**g**) Open-circuit voltage (Voc), short-circuit current (Isc), and transfer charge (Qsc) of TW120-TENGs at different working displacements. (**h**) The 10,000 contact-separation cycles of TW120-TENG.

To investigate the effect of the degree of delignification on electrical output performance, TW-TENGs based on different delignification times were connected to an electrostatic meter and a tensile tester, respectively, and contact-separation motions were performed in single-electrode mode with PDMS film as another tribolayer to generate electrical output, which was compared with that of NW-TENGs with logs as the tribolayer. The tensile tester was operated to set the frequency of the contact-separation motion to 1 Hz, the working displacement to 5 mm, and the pressure to 10 N for the electrical output performance test of the TW-TENGs. As shown in Figure 4a, the electrical output performance of the TW-TENGs was significantly improved compared to that of NW-TENG, especially the open-circuit voltage (Voc) of TW120-TENG which reached up to ~127 V, which was 6.3 times higher than that of NW-TENG, and the Voc of TW120-TENG increased by ~62.8% compared to that of TW0-TENG. The significant improvement is due to the fact that the successful removal of lignin with antistatic properties and the introduction of strong electron-donating groups (amino and methyl) in the epoxy resin [40,41,42]. Subsequently, the electrical outputs of TW150-TENG and TW180-TENG decreased slightly, probably due to the excessive delignification treatment that disrupted the integrity of the wood templates and hindered the impregnation of epoxy resin, which in turn weakened the triboelectrical properties of TENGs.

Working frequency and working displacement are important factors that affect the electrical output performance of TENGs. We explored the open-circuit voltage (Voc), short-circuit current (Isc), and transfer charge (Qsc) of T120-TENG versus working frequency and working displacement (Figure 4b–g) [43,44,45]. The working displacement of the contact-separation motion was set as 5 mm and the working pressure was set as 10 N. Figure 4b–d show the corresponding Voc, Isc, and Qsc of TW120-TENG at different working frequencies (0.5~2.5 Hz). As shown, with the gradual increase in working frequency, the Voc and Qsc stabilized at ~127 V and ~80 nC, respectively, with them being largely unaffected by the working frequency. However, when the working frequency increased from 0.5 Hz to 2.5 Hz, the Isc increased from ~200 nA to ~1221 nA, an increase of ~512%. This enhancement arises from the correlation between the elevated working frequency and heightened inrush frequency, resulting in an accelerated transfer rate of external electrons, shortened transfer periods, and diminished durations of current peaks, which collectively contribute to the increase in the Isc, while the Voc and Qsc mainly depend on the electron-gaining/donating ability of the triboelectric material of TW-TENG itself and its own structural design, so changes in working frequency have no significant effect on them [46]. Figure 4e–g demonstrates the relationship between working displacement and electrical output performance. The Voc, Isc, and Qsc generated by the TW120-TENG under 1~5 mm variation of working displacement were tested by fixing the working frequency at 1 Hz and the working pressure at 10 N. From the data, it can be seen that as the working displacement increases, the Voc of the TW120-TENG grows from ~68 V to ~127 V, an increase of ~86.8%, the Isc grows from ~100 nA to ~500 nA, an increase of 400%, and the Qsc grows from ~43 nC to ~112 nC, an increase of 160.5%, which is mainly attributed to the subsequent increase in the potential difference between the tribopositive and tribonegative materials when the working displacement increases, which in turn strengthens the triboelectrical properties of the TW-TENGs, leading to greater electrical output [47].

We investigated the electrical output of TW-TENGs with different working pressures (1, 5, 10, 20, and 30 N) and different sizes (3 × 3 cm^2^, 4 × 4 cm^2^, 5 × 5 cm^2^) to evaluate the triboelectric properties of TW120-TENG more comprehensively (Figures S4 and S5). The Voc of the TW-TENGs was positively correlated with the working pressure, which increased by 122% from ~68 V to ~151 V when the working pressure increased from 1 N to 30 N. This was ascribed to the larger working pressure, which facilitated closer surface contact between the two tribolayers. The Voc of the TW-TENGs changed with the size of the tribomaterial from 3 × 3 cm^2^ to 5 × 5 cm^2^ and increased significantly from ~74 V to ~205 V, which is attributed to there being more triboelectric charge due to the larger contact size. The durability and stability of TW-TENGs in long-cycle operation are particularly important for their practical application; hence, we conducted durability tests on the TW-TENG (Figure 4h). Under the working conditions of 10 N, 1 Hz, and 5 mm, the Voc of the TW120-TENG basically remained stable after 10,000 cycles without significant degradation, proving the electrical stability of TW-TENGs.

**Figure 5:**
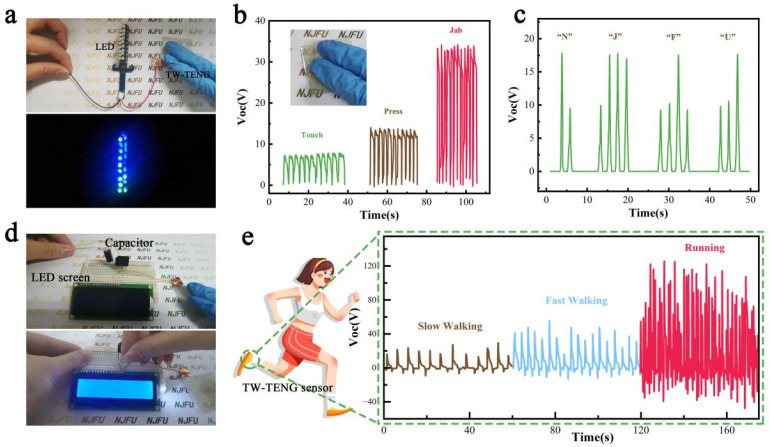
Demonstration of the practical application of the TW-TENG. (**a**) The TW-TENG lights up 19 small LED bulbs. (**b**) Open-circuit voltage (Voc) is generated by triggering the TW-TENG in different motion modes. (**c**) The TW-TENG transmits the message of “NJFU” in Morse code. (**d**) The TW-TENG charges a capacitor and lights up an LED screen with the capacitor as the power source. (**e**) The TW-TENG serves as a motion sensor to monitor human movement.

To demonstrate the applicability of our TW-TENGs in real applications, we connected TW120-TENG to 19 commercial LED bulbs, and all of the bulbs were successfully lit up by pressing the TW120-TENG with only two fingers (Figure 5a), eliminating the dependence on an external power supply. In order to truly demonstrate the electrical output of the TW-TENGs in daily life scenarios, the electrical signals generated by different hand movements, i.e., “Touch”, “Press”, and “Jab”, were tested by connecting TW120-TENG to an electrostatic meter. As shown in Figure 5b, the Voc from “touch” is about 7~8 V, the Voc from “press” is about 17~19 V, and the pressure of “Jab” increases significantly, and the Voc increases to 32~34 V. To further test the accuracy of TW-TENGs as a sensor, an attempt was made to control the voltage magnitude by varying the magnitude of pressure, with higher voltage (~17 V) representing the long signal and lower voltage (~8 V) representing the point signal to simulate Morse code. The deliberately controlled clicks of TW120-TENG successfully transmitted the message “NJFU”, as shown in Figure 5c. In addition to being used directly to power electronic devices, the power generated by TW-TENGs can also be stored in a capacitor for future use. Figure 5d shows TW120-TENG being used to charge a commercial capacitor, and the charged capacitor can successfully light up a little LED screen. To further verify the utility of TW-TENG as a wearable sensor, TW120-TENG was attached to the sole of the foot to monitor the electrical output when a person is walking and running, as shown in Figure 5e. During walking and running, the human foot makes a regular contact separation movement with the ground, so the TENG outputs regular voltage signals. The voltage signals excited by the motion modes of slow walking, fast walking, and running are all significantly different, and the output of the TW-TENG is gradually enhanced by the working pressure and frequency caused by the motion modes, which is in line with the law obtained from the analysis of the working conditions of the TW-TENG above.

## 4. Conclusions

In this study, a transparent wood-based triboelectric nanogenerator (TW-TENG) is innovatively proposed, combining high output performance, excellent light transmittance, and wood aesthetics. The efficient TW-TENG was successfully prepared by delignifying NW and impregnating epoxy resin with strong electron-donating groups and a refractive index that is congruent with that of wood. TW120-TENG outputs an open-circuit voltage of up to ~127 V, a short-circuit current of up to ~966 nA, and a transfer charge of up to ~80 nC, and higher electrical outputs can be obtained by controlling the working conditions and the size of TW-TENGs. In addition to its excellent optical properties, with an optical transmittance of up to 88.8%, the TW-TENG also exhibits excellent flexibility and hydrophobicity. Furthermore, different degrees of delignification endow TW-TENGs with different colors, demonstrating high aesthetic value and potential for smart decorative materials. Finally, we demonstrate the electrical signals output from TW-TENGs in different scenarios or motion modes, proving the application prospects of TW-TENGs in the fields of mechanical energy harvesting, motion sensors, and smart home appliances.

## Data Availability

The original contributions presented in the study are included in the article/Supplementary Materials, further inquiries can be directed to the corresponding authors.

## Supplementary Materials

The following supporting information can be downloaded at: https://www.mdpi.com/article/10.3390/polym16091208/s1, Figure S1. Morphological characterization of TW: (a) cross-sectional SEM images of TW90; (b) cross-sectional SEM images of TW150; (c) cross-sectional SEM images of TW180; Figure S2. Tensile strength and elongation at break of the DW with different degrees of delignification; Figure S3. Stress–strain curves of the DW with different degrees of delignification; Figure S4. Open-circuit voltage of the TW-TENG at different working forces; Figure S5. Open-circuit voltage of the TW-TENG of different sizes.
