# Supplementary material for: Transparent and Efficient Wood-Based Triboelectric Nanogenerators for Energy Harvesting and Self-Powered Sensing
Source: Polymers (Basel). 2024 Apr 26;16(9):1208. doi: 10.3390/polym16091208 (PMC11085067; doi:10.3390/polym16091208)
Supplement: Supplementary file 1 [file polymers-16-01208-s001.zip › polymers-2941945-supplementary.pdf]

Supplementary Materials

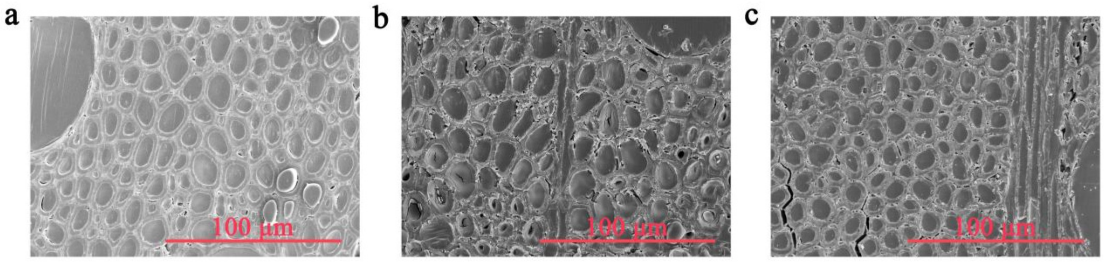

Figure S1. Morphological characterization of TWs. (a) Cross-sectional SEM images of TW90. (b) Cross-sectional SEM images of TW150; (c) Cross-sectional SEM images of TW180.

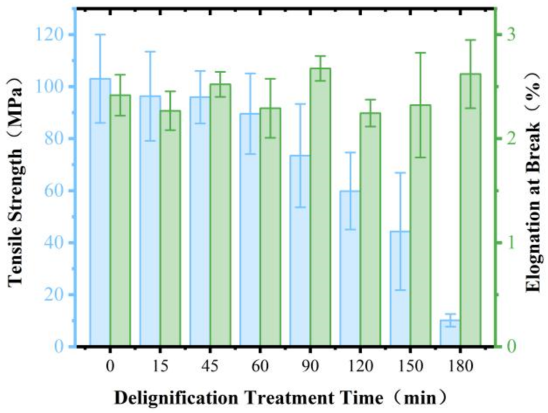

Figure S2. Tensile strength and elongation at break of DW with different degrees of delignification.

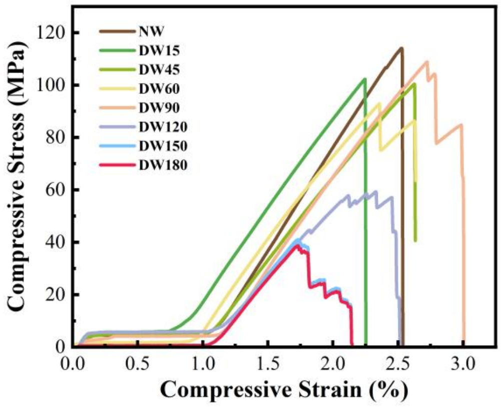

Figure S3. Stress-strain curves of DW with different degrees of delignification.

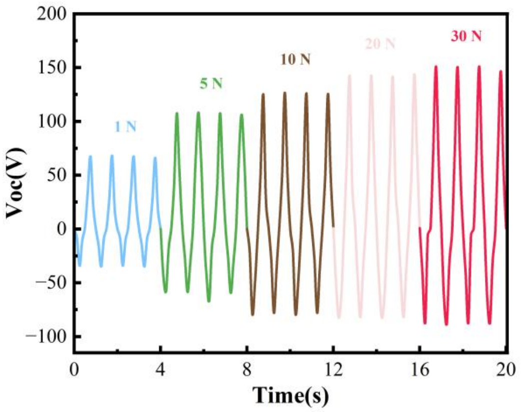

Figure S4. Open-circuit voltage of TW-TENG at different working forces.

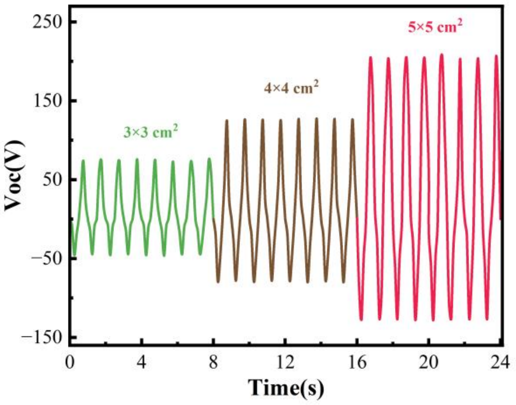

Figure S5. Open-circuit voltage of TW-TENG of different sizes.
